# Recognizing, managing, and preventing cognitive sequelae after stroke. A narrative review

**DOI:** 10.1016/j.cccb.2026.100543

**Published:** 2026-04-08

**Authors:** Leonardo Pantoni, Slaven Pikija, Tuyen Van Nguyen, Michael Brainin

**Affiliations:** a Neuroscience Research Center, Department of Biomedical and Clinical Sciences “L. Sacco”, University of Milan, Via Giovanni Battista Grassi 74, 20157 Milan (MI), Italy; b Department of Neurorehabilitation Sciences, Casa di Cura Igea, Via Marcona 69, 20129 Milan (MI), Italy; c Department of Neurology, Medical University of Graz, Neue Stiftingtalstrasse 6, 8036 Graz, Austria; d Department of Neurology, Paracelsus Medical University Salzburg, Strubergasse 21, 5020 Salzburg, Austria; e Stroke Center, 108 Military Central Hospital, No. 1 Tran Hung Dao Street, Bach Dang Ward, Hai Ba Trung District, Hanoi, 100000, Vietnam; f Department of Clinical Neurosciences and Preventive Medicine, Danube University Krems, Steiner Landstrasse 124, 3500 Krems an der Donau, Austria

**Keywords:** Post-stroke cognitive impairment, Vascular cognitive impairment, Post-stroke dementia, Cerebral small vessel disease, Pathophysiology, Biomarkers, Prevention

## Abstract

Post-stroke cognitive impairment (PSCI) affects up to one-third of stroke survivors and is a major contributor to long-term disability and reduced quality of life. Its heterogeneous phenotype reflects the interplay of acute cerebrovascular injury with chronic vascular pathology, inflammation, blood–brain barrier dysfunction, and, in many patients, coexisting neurodegeneration. Clinical manifestations vary according to lesion location and most commonly involve executive and attentional deficits, with variable impairment of memory, language, visuospatial function, and neuropsychiatric domains. Cognitive trajectories range from partial early recovery to persistent or progressive decline.

Diagnosis remains challenging because covert cerebrovascular disease and overlapping neurodegenerative processes may mimic or amplify deficits. A stepwise diagnostic strategy combining focused cognitive screening, neuropsychological assessment when indicated, and neuroimaging is therefore recommended. In many acute stroke settings, CT continues to serve as the routine first-line modality, whereas MRI allows more detailed characterization where available. Emerging adjuncts include fluid biomarkers of neuroaxonal injury, inflammation, and vascular brain injury, as well as digital and telephone-based tools that may support longitudinal monitoring in selected populations.

Treatment options remain limited. Non-pharmacological interventions, including cognitive rehabilitation, physical activity, and multimodal neurorehabilitation, are central to current management, although PSCI-specific high-quality evidence is scarce. Prevention is essential and should focus on vascular risk-factor control, lifestyle modification, cognitive reserve, and social engagement.

This narrative review synthesizes current evidence on the mechanisms, presentation, diagnosis, and management of PSCI and highlights priorities for harmonized care pathways and the routine integration of cognitive health into stroke care.

## Highlights

• Cognitive deficits after stroke are common and often underrecognized.
• Executive dysfunction and attentional deficits predominate clinically.
• Small vessel disease and atrial fibrillation promote progressive cognitive decline.
• Neuroimaging plus biomarkers and digital tests support assessment and monitoring.
• Early cognitive rehabilitation and exercise plus risk-factor control are central.

## 1 Introduction

Post-stroke cognitive impairment (PSCI) is a common and heterogeneous complication of stroke, with prevalence estimates varying substantially depending on the definitions used, the cognitive domains assessed, the instruments applied, and the time of evaluation. Across the spectrum from subtle domain-specific deficits to overt dementia, PSCI is a major contributor to long-term disability, reduced quality of life, and loss of functional independence [1,2]. Its societal relevance is further amplified by the growing population of stroke survivors, including younger adults, in whom cognitive sequelae may substantially affect return to work and long-term social participation [[3], [4], [5]]. Although much of the available mechanistic and longitudinal evidence derives from ischemic stroke cohorts, the broader clinical construct of PSCI is not confined to ischemic stroke and also includes cognitive impairment after hemorrhagic stroke [6].

Cognitive trajectories after stroke are dynamic rather than uniform. While some patients—particularly after mild to moderate stroke—show partial improvement during the first weeks to three months, recovery is often incomplete, and a substantial proportion develop persistent impairment or later progressive decline [[7], [8], [9], [10]]. PSCI should therefore be understood not as a single static syndrome, but as a heterogenous and evolving clinical entity.

The clinical presentation of PSCI is equally heterogeneous, often affecting attention, memory, executive function, language, and visuospatial abilities [11]. This heterogeneity can be understood as the result of interactions between pre-stroke vulnerability, characteristics of the index stroke, and biological processes that evolve after the acute event, consistent with prior multidimensional PSCI frameworks [6]. Relevant contributors include baseline brain health, cognitive reserve, covert or overt neurodegenerative pathology, lesion location and extent, and secondary mechanisms that may shape subsequent cognitive trajectories [11,12]. Together, these factors help explain the marked variability in cognitive outcomes after stroke.

A further challenge lies in distinguishing stroke-related cognitive decline from pre-existing or mixed pathologies. This is particularly relevant in older patients, in whom covert cerebrovascular disease, small vessel disease, silent infarcts, and coexisting neurodegenerative processes may influence both presentation and prognosis [13,14]. Given the growing burden of long-term cognitive sequelae after stroke, a more integrated understanding of PSCI is needed. This review therefore synthesizes current evidence on its pathophysiology, clinical presentation, diagnostic challenges, and therapeutic approaches, while highlighting key unmet needs in research and clinical practice.

## 2 Pathophysiological mechanisms of PSCI

Post-stroke cognitive impairment results from the interaction of pre-existing brain vulnerability, lesion characteristics and network disruption, and post-stroke vascular, inflammatory, and neurodegenerative processes [15]. Understanding these pathophysiological processes is essential for risk stratification, targeted prevention, and the development of disease-modifying interventions. The presentation and severity of cognitive deficits are influenced not only by the characteristics of the index stroke, including location, volume, and type, but also by pre-existing cerebrovascular and neurodegenerative conditions that may interact synergistically to accelerate cognitive decline. Furthermore, the underlying mechanisms vary across populations, reflecting differences in genetic susceptibility, vascular risk-factor profiles, and environmental exposures. This chapter examines the key pathophysiological pathways contributing to PSCI, from acute ischemic injury and chronic small vessel disease to the broader neurodegenerative processes that often coexist with cerebrovascular pathology.

### 2.1 Acute ischemic injury and strategic lesions

The most immediate contributor to PSCI is focal ischemic injury, causing neuronal death and disconnection within critical cognitive networks. The impact of tissue loss on cognition depends not only on its extent but primarily on its location. Strategic regions such as the thalamus, hippocampus, and basal ganglia are particularly relevant, with even small lesions in these areas capable of producing substantial cognitive deficits [11]. The stroke subtype further shapes cognitive outcomes: large cortical infarcts tend to result in domain-specific deficits, whereas strokes due to small vessel disease are associated with more diffuse, subcortical impairment, particularly affecting attention, processing speed, and executive function [[16], [17], [18]].

In many patients, the acute infarct acts as an inflection point superimposed on pre-existing brain vulnerability. Following this event, cognitive trajectories may diverge toward partial recovery, persistent impairment, or later decline depending on lesion characteristics, baseline reserve, concurrent small vessel disease, and coexisting neurodegenerative pathology [7]. This perspective helps reconcile the coexistence of early domain-specific improvement in some patients with later deterioration in others [9,10].

### 2.2 Chronic mechanisms

#### 2.2.1 Inflammation and blood-brain barrier dysfunction

As PSCI progresses into the subacute and chronic phases, long-term processes become increasingly important. Ischemic injury initiates an immune response involving microglial and astrocyte activation and the release of pro-inflammatory cytokines [19]. While initially cerebroprotective, chronic inflammation can cause secondary neuronal injury and disrupt synaptic plasticity, even in brain regions remote from the primary infarct, thereby promoting cognitive decline [15,19]. Persistent blood-brain barrier dysfunction may further exacerbate these processes by facilitating entry of peripheral immune mediators and impairing clearance of metabolic waste [15].

Stroke complications should also be considered within this mechanistic framework. Infection—particularly pneumonia and urinary tract infection—is common after stroke and has been associated with both acute and medium-term PSCI [20]. This relationship is likely mediated by a combination of systemic inflammatory stress, blood-brain barrier vulnerability, secondary neuronal injury, and reduced physiological reserve [6,15,20]. Clinically, this is especially relevant because infection prevention and early treatment may represent tractable strategies for reducing cognitive morbidity after stroke [20].

#### 2.2.2 Vascular pathology: small vessel disease and atrial fibrillation

Of the chronic vascular mechanisms, small vessel disease is a key factor in post-stroke cognitive decline, particularly in individuals with vascular risk factors such as hypertension or diabetes [16,21]. Small vessel disease affects the small arteries, arterioles, venules, and capillaries of the brain [16]. It encompasses a spectrum of pathological changes, including white matter hyperintensities, lacunes, and cerebral microbleeds, which contribute to cognitive impairment and dementia [1,21]. These chronic mechanisms involve age-dependent processes, including arterial stiffness, vascular oxidative stress, and endothelial dysfunction [22].

Atrial fibrillation is another plausible contributor to post-stroke cognitive decline, but the independence of this association remains difficult to establish consistently across studies [23,24]. Proposed mechanisms include recurrent overt or covert embolic injury, chronic cerebral hypoperfusion, and interaction with coexisting small vessel disease. However, observed associations may be influenced by stroke severity, recurrent stroke burden, and underlying vascular brain injury. Atrial fibrillation should therefore be viewed as an important marker of vascular brain vulnerability and recurrent embolic risk, while specific pathways linking atrial fibrillation to PSCI require further clarification.

### 2.3 Interaction with neurodegeneration, reserve, resilience, resistance, frailty, and genetics

Ischemic injury can accelerate amyloid-β aggregation [25] and tau-related neurodegenerative processes [26], promoting mixed vascular and neurodegenerative pathology that complicates diagnosis and treatment [25]. In this context, the concepts of reserve, resilience, and resistance help explain the marked interindividual variability in post-stroke cognitive outcomes.

Cognitive reserve refers to the brain’s latent ability to tolerate structural damage without overt clinical manifestation [[27], [28], [29]]. It therefore refers to the capacity to tolerate or compensate for existing brain pathology without immediate clinical expression, whereas resistance describes the ability to limit the development of such pathology in the first place despite exposure to risk. It is shaped across the lifespan by educational level, occupational complexity, and cognitively stimulating activities [27,30]. Cognitive resilience reflects the dynamic capacity of the brain to adapt to acute and chronic insults in real time through plasticity, network reorganization, and the recruitment of compensatory pathways [28,29].

Although conceptually distinct, these constructs overlap with related notions such as brain reserve, brain resilience, compensation, and frailty. Whereas brain reserve and brain resilience generally refer more to structural or biological capacity, cognitive reserve and cognitive resilience describe the ability to maintain cognitive performance despite existing brain pathology or injury. This broader conceptual vocabulary is relevant in PSCI because patients with apparently similar infarct burden or white matter injury may experience substantially different cognitive trajectories and responses to rehabilitation [[31], [32], [33]]. Recovery is also influenced by clinical factors such as older age, diabetes mellitus, lesion burden and location, and early executive or other domain-specific deficits, all of which have been associated with less favorable cognitive outcome [10,34].

Frailty describes a state of reduced physiological reserve and heightened vulnerability to stressors, which may adversely influence recovery and cognitive outcomes after stroke. It is particularly relevant in older stroke populations, where recent data suggest that pre-stroke frailty predicts post-stroke neurocognitive disorder more strongly than pre-stroke dependency alone, underscoring the importance of whole-patient vulnerability rather than stroke severity in isolation [35]. Recent work further suggests that frailty may be most informative when considered together with pre-stroke cognitive status, as a combined frailty-plus-cognition framework may better support risk stratification, treatment selection, and rehabilitation planning in stroke populations [36]. In an aging stroke population, frailty should therefore be considered alongside reserve and resilience in both prognostic assessment and rehabilitation planning.

Genetic susceptibility may also contribute to PSCI heterogeneity by influencing neuroplasticity, inflammatory signaling, susceptibility to small vessel disease, and the interaction between cerebrovascular and neurodegenerative processes. Among the most plausible candidates, brain-derived neurotrophic factor (BDNF)-related pathways have been associated with cognitive recovery after stroke [37], whereas apolipoprotein E (APOE) remains of interest because of its established links to Alzheimer-type neurodegeneration, although evidence for an independent effect in PSCI remains inconsistent [38,39]. More exploratory findings have implicated circadian genes and related pathways, but these associations require further replication [40]. At present, genetic markers are best viewed as contributors to biological heterogeneity and recovery potential rather than as stand-alone tools for routine PSCI stratification [41].

## 3 Clinical presentation of PSCI

Post-stroke cognitive impairment presents with a heterogeneous range of deficits, determined by lesion characteristics, pre-stroke cognitive status, and vascular risk factors. Symptoms usually emerge acutely but may also develop or worsen over time. While some patients experience partial recovery, persistent impairments are common, significantly interfering with daily functioning and reducing quality of life [2].

### 3.1 Core features: executive dysfunction and attentional deficits

Executive dysfunction and attentional deficits are among the most prevalent features of PSCI, often more pronounced than memory impairment [42]. These deficits typically result from damage to frontal-subcortical networks [42] and manifest as problems with planning, shifting attention, and problem-solving [43,44]—abilities that are essential for independent living [43] and participating in and adhering to rehabilitation programs [44,45].

### 3.2 Other cognitive domains

Memory impairment is also frequent but generally reflects challenges with attention and executive function rather than a classical hippocampal amnesia [42,46]. Patients may struggle with encoding and retrieving information, particularly in complex or distracting environments [42,47]. This pattern is consistent with current vascular cognitive impairment guidance, where memory difficulties are often characterized by inefficient encoding and/or retrieval rather than impaired consolidation typical of Alzheimer’s disease [48].

Language and visuospatial deficits are often contingent on lesion location. Language dysfunction, including aphasia, is common after left hemispheric strokes and can have long-term consequences for communication and social participation. In contrast, right hemispheric lesions are more likely to cause spatial attention deficits, particularly hemispatial neglect, leading to difficulties with spatial awareness and visuospatial processing [11].

### 3.3 Common comorbidities: psychiatric symptoms

Psychiatric symptoms such as depression, anxiety, apathy, and emotional lability frequently accompany cognitive deficits [2]. These symptoms can exacerbate perceived impairment and reduce motivation for rehabilitation, thereby further compromising functional outcomes [49]. Routine screening and integrated management of these mood and motivational disturbances remain important because they directly influence functional recovery and overall quality of life [50].

Diagnostic heterogeneity in PSCI is further complicated by speech disturbances such as aphasia and coexisting affective disorders such as depression, which can obscure test performance and mimic or exacerbate cognitive impairment [6]. Neuropsychiatric symptoms should be actively assessed and addressed as part of comprehensive post-stroke cognitive care, because depression, apathy, anxiety, fatigue, and sleep-related problems may interact with cognitive deficits and functional recovery. Interdisciplinary management is therefore important when PSCI is suspected or established [6].

### 3.4 Diagnostic limitations in routine clinical practice

Despite the clinical importance of these diverse presentations, accurate and early identification requires systematic evaluation across all cognitive domains. In routine stroke care, however, cognitive screening remains inconsistently implemented, and many patients remain unassessed unless they exhibit overt behavioral changes or self-report difficulties. Although standardized neuropsychological testing remains the diagnostic gold standard, its use is often limited in practice for reasons including resource constraints, time demands, patient fatigue, and limited attention to cognitive assessment in routine stroke services [6,51,52]. Importantly, understanding domain-specific patterns in PSCI is essential not only for accurate diagnosis, but also for prognosis and the development of individualized interventions.

## 4 Structured and emerging approaches to PSCI diagnosis

The accurate and timely diagnosis of PSCI remains a central challenge, necessitating a multi-step assessment approach. Current diagnostic pathways are often hindered by variable screening practices [6,52], limited access to formal neuropsychological assessment [53], and incomplete characterization of the underlying brain pathology in routine clinical care [54,55].

### 4.1 The multi-step approach to clinical assessment

Accurate and timely diagnosis of PSCI requires a structured, multi-step approach. For initial cognitive screening, the Montreal Cognitive Assessment (MoCA) is a useful option in post-acute stroke settings and may be more sensitive than the Mini-Mental State Examination (MMSE) for milder deficits; however, it should not replace comprehensive clinical or neuropsychological assessment and may be less feasible in patients with aphasia, neglect, or visual impairment [6,52].

Comprehensive neuropsychological assessment remains the reference standard for defining cognitive profiles after stroke. It is particularly valuable in patients with persistent complaints, unexplained functional difficulties, or discordant screening results. In more typical presentations, such detailed assessment may help identify patterns that are more suggestive of a predominantly vascular or predominantly Alzheimer-type process, but in individual patients—especially older adults with mixed pathology—this distinction often remains challenging and should not be overstated.

In addition to direct cognitive testing, informant-based tools such as the Informant Questionnaire on Cognitive Decline in the Elderly (IQCODE) can provide valuable baseline information on pre-stroke cognition. They are particularly helpful when direct testing is unreliable, such as during the acute stroke phase, and may support the identification of pre-existing cognitive decline [56,57].

Timing is a key component of structured PSCI assessment. Targeted cognitive assessment in the subacute phase may help identify patients who warrant closer follow-up, comprehensive neuropsychological evaluation, and individualized rehabilitation planning. However, current guidance does not support indiscriminate routine screening of all stroke survivors, and the optimal timing and frequency of assessment remain insufficiently defined [6,52]. Reassessment at standardized intervals, such as 3, 6, and 12 months, may improve detection of persistent, delayed, or progressive impairment and refine individualized management over time [[6], [7], [8], [9], [10]].

In the acute and subacute setting, PSCI should also be distinguished from other causes of fluctuating or newly apparent cognitive dysfunction, particularly delirium, recurrent stroke, metabolic or infectious complications, and post-stroke recrudescence. Delirium is common after stroke and may mimic or obscure evolving PSCI, while post-stroke recrudescence can cause transient re-emergence of prior deficits in the absence of a new infarct [6,58]. Distinguishing these entities is essential for accurate diagnosis, appropriate work-up, and longitudinal management [59].

In selected stroke populations, early domain-specific neuropsychological testing can provide clinically useful additional detail, particularly when screening findings, patient-reported symptoms, and functional performance do not align fully [34,60]. In this context, selected adjunctive tools may support standardization and longitudinal follow-up in appropriate patients, but they should be regarded as complements rather than replacement for clinician-guided evaluation. Older adults and patients with aphasia, neglect, visual impairment, motor limitations, or low digital literacy may complete self-administered assessments less reliably, and in severe impairment, detailed testing—digital or otherwise—may be neither feasible nor clinically necessary [61,62].

### 4.2 The role of neuroimaging

Neuroimaging plays a critical complementary role in the diagnosis and risk stratification of PSCI. Many stroke survivors harbor covert cerebrovascular pathology that may not be clinically apparent but significantly influences cognitive trajectories and response to therapy. Computed tomography (CT) remains the standard first-line imaging modality in acute stroke care across many settings, including both low- and middle-income countries and well-resourced health systems. Magnetic resonance imaging (MRI), however, provides more detailed characterization of strategic infarcts, white matter hyperintensities, lacunes, cerebral microbleeds, and other structural markers relevant to PSCI, and is therefore particularly informative when a more refined assessment of structural contributors to cognitive impairment is needed [63,64]. Even so, guideline support for the routine use of imaging markers for cognitive prognostication remains limited, with the clearest signal relating to substantial white matter hyperintensities on MRI [48,52]. At the same time, clinically acquired CT may still help identify patients at increased risk of persistent PSCI, particularly in domains such as memory and attention, although this should currently be interpreted as observational evidence rather than as support for routine CT-based cognitive prognostication [65].

### 4.3 Future directions: Biomarkers and digital tools

The diagnostic and monitoring landscape for PSCI is evolving toward a multimodal, risk-stratified approach that integrates fluid biomarkers and scalable cognitive tools.

Neurofilament light chain remains one of the most promising markers of neuroaxonal injury, while tau, glial fibrillary acidic protein (GFAP), and inflammatory markers are also under investigation [[66], [67], [68]].

An important translational example in vascular cognitive research is the NIH-funded Biomarkers for Vascular Contributions to Cognitive Impairment and Dementia (MarkVCID) consortium, which was established to identify and validate imaging and fluid biomarkers for small-vessel-disease-related vascular contributions to cognitive impairment and dementia. Although not specific to PSCI, this framework is highly relevant because it illustrates how harmonized, multicenter biomarker pipelines may improve risk stratification and trial readiness in vascular cognitive disorders more broadly [69]. Candidate biomarkers prioritized for further validation within the MarkVCID framework include imaging- and physiology-based measures such as peak width of skeletonized mean diffusivity, free water, MRI markers of arteriosclerosis, and cerebrovascular reactivity, together with selected blood-based markers such as neurofilament light chain, thereby illustrating the type of multimodal panel that may ultimately support more biologically informed stratification and harmonized clinical trial design in vascular cognitive disorders.

In parallel, digital and remote tools represent a potentially important advance in PSCI assessment and longitudinal monitoring. In appropriate patients, these approaches may enhance broader clinical applicability, standardization, and longitudinal follow-up, and may be particularly useful when in-person assessment is impractical.

Telephone-based instruments such as the modified Telephone Interview for Cognitive Status (TICSm) and the telephone MoCA may support longitudinal surveillance when face-to-face evaluation is not feasible [62]. However, implementation requires caution: digital versions of cognitive tests cannot be assumed to share the same reliability, validity, or normative framework as their paper counterparts, and limited digital access or literacy may exacerbate disparities in vulnerable groups [61]. These methods should therefore be validated in stroke-specific populations and interpreted within a broader clinical context.

Looking further ahead, digital assessment may increasingly expand beyond structured test administration toward passive digital phenotyping, involving the continuous, unobtrusive collection of data from personal devices such as smartphones and wearables. This may enable quantification of real-world functional and behavioral markers, including day-to-day variability in activity levels, sleep–wake regularity, mobility patterns, and communication rhythms, thereby complementing standard neuropsychological assessments. Realizing this potential, however, will require careful attention to device variability, platform-specific norms, data interpretation, and equitable access [61].

Ultimately, a harmonized, multi-step pathway that combines targeted cognitive assessment, comprehensive neuropsychological evaluation when indicated, appropriate neuroimaging, and selected digital or biomarker-based adjuncts may offer a robust route to earlier detection and longitudinal monitoring while preserving essential clinical context [6,48,52,62,69].

### 4.4 Global perspectives: ethnic and regional variability in PSCI

Understanding ethnic and regional variability in PSCI is essential for accurate diagnosis, appropriate risk assessment, and effective clinical management. Differences across ethnoracial and regional groups likely reflect variation in vascular risk burden, educational background, and the effects of established risk factors on cognition, rather than biology alone [6,70]. Population-based U.S. data, including the REGARDS study (Reasons for Geographic and Racial Differences in Stroke), further suggest that post-stroke cognitive decline may be greater in Black than in White stroke survivors [6,71]. As the field moves toward more individualized diagnostic strategies, equitable access to accurate PSCI detection and comprehensive post-stroke support across diverse populations should remain a central priority [6].

## 5 Current and emerging approaches to treating PSCI

The management of PSCI remains a significant challenge in clinical care. This is largely due to the condition’s heterogeneity, the absence of specifically approved pharmacological agents, and the insufficient integration of cognitive management into routine stroke care pathways [64,72]. While awareness of the cognitive burden following stroke is growing, the implementation of targeted interventions remains inconsistent.

### 5.1 Pharmacological strategies in PSCI: a developing field

To date, pharmacological approaches have primarily focused on symptomatic relief or on repurposing agents that influence vascular or neurodegenerative mechanisms. More recently, there has been growing recognition of the need for disease-modifying and neurorestorative compounds targeting underlying pathophysiological processes such as inflammation, oxidative stress, synaptic dysfunction, and impaired neuroplasticity. Nevertheless, robust PSCI-specific evidence remains limited, and no drug has yet demonstrated clear efficacy sufficient for routine use [6,52,73].

An important example of ongoing research is the CODEC study, a randomized, placebo-controlled, double-blind trial designed to assess the efficacy and safety of Cerebrolysin in post-stroke cognitive decline (ISRCTN88122184) [74]. Cerebrolysin is hypothesized to exert pleiotropic neurorestorative effects relevant to PSCI, including support of neuroplasticity and attenuation of secondary injury pathways. This trial is therefore particularly important because it uses a PSCI-focused design with long-term cognitive follow-up.

Complementing the randomized evidence, the CREGS study (Cerebrolysin REGistry study in Stroke) provided multinational, high-quality comparative effectiveness data in approximately 1800 patients with moderate acute ischemic stroke (NIHSS 8–15) [75]. Cognitive outcomes favored Cerebrolysin in this real-world setting, with higher 90-day MoCA scores in the treated group across the overall target population and an even more pronounced effect among patients with evidence of pre-stroke cognitive decline based on baseline IQCODE (>3.3).

Other pharmacological strategies remain exploratory; cholinesterase inhibitors such as donepezil and N-methyl-d-aspartate (NMDA) receptor antagonists such as memantine have shown at most modest symptomatic benefit in some vascular or post-stroke cognitive impairment populations, but the evidence remains inconsistent and insufficient to support routine use specifically for PSCI [52,76]. Taken together, current evidence highlights the need for methodologically robust, PSCI-specific trials with standardized cognitive endpoints and longer follow-up to more reliably evaluate pharmacological strategies and guide future evidence-based practice.

### 5.2 Non-pharmacological interventions: central components of current practical management

Currently, non-pharmacological interventions represent an important practical component of PSCI management, although the strength of PSCI-specific evidence varies across intervention types and remains limited overall [6,52]. A growing body of evidence highlights the benefits of cognitive rehabilitation [77], physical exercise [78], and innovative neurorehabilitation technologies [79]. Multidomain programs that combine lifestyle modification with vascular risk-factor management have also been tested in the post-stroke setting [77,80]. For instance, the randomized Austrian Polyintervention Study to Prevent Cognitive Decline after Ischemic Stroke (ASPIS) trial evaluated a 24-month multifactorial lifestyle intervention initiated within three months post-stroke. However, the intervention did not significantly reduce cognitive decline compared with standard care; this neutral result may partly reflect the predominantly mild-stroke population, the lower-than-expected event rates, and limited power to detect small differences, highlighting the continuous need for adequately powered PSCI-specific trials [80].

#### 5.2.1 Cognitive and physical rehabilitation

Structured cognitive training programs, such as therapist-guided computerized exercises, may provide significant benefits when initiated early after stroke [81]. Likewise, physical activity—particularly combined aerobic and strength training—has been associated with improved cognitive outcomes, with measurable effects reported even in the chronic phase after stroke [78]. Such interventions may enhance attention and processing speed and may also support cognition indirectly through improvements in mood, sleep, and overall vitality [78,82]. Whether these short-term cognitive benefits translate into sustained gains in functional independence and quality of life remains to be clarified in high-quality studies [83].

#### 5.2.2 Multimodal and technological approaches

**Fig. 1:**
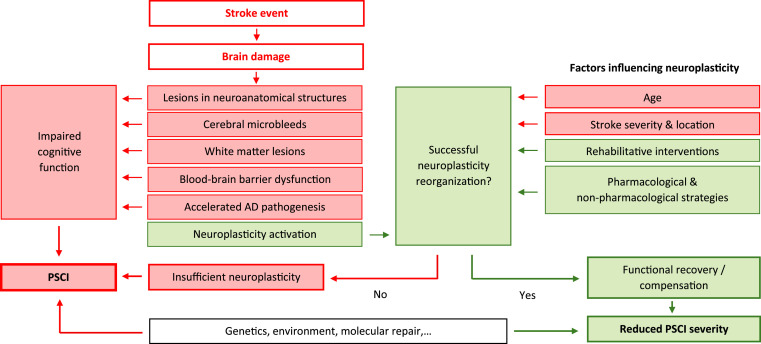
Neuroplasticity as a key determinant of cognitive outcome after stroke. Schematic overview showing how a stroke event leads to brain damage with structural and vascular changes (lesions in neuroanatomical structures, cerebral microbleeds, white matter lesions, and blood-brain barrier dysfunction) and may accelerate Alzheimer-type pathogenesis, contributing to impaired cognitive function and post-stroke cognitive impairment. In parallel, brain damage triggers neuroplasticity activation. Cognitive trajectory depends on whether successful neuroplastic reorganization occurs: successful reorganization supports functional recovery/compensation and reduced severity of post-stroke cognitive impairment, whereas failure results in insufficient neuroplasticity and persistent cognitive impairment. Factors influencing neuroplastic reorganization include age, stroke severity and location, rehabilitative interventions, and pharmacological and non-pharmacological strategies; genetic and environmental factors and molecular repair processes may also modulate recovery.

*Abbreviations:* AD, Alzheimer’s disease; BBB, blood-brain barrier; PSCI, post-stroke cognitive impairment.

Effective management of PSCI increasingly requires addressing the full range of contributing factors and moving beyond the traditional focus on motor deficits in stroke rehabilitation [84]. Multimodal interventions are therefore gaining importance. Available evidence suggests that combining cognitive and physical rehabilitation approaches may yield greater benefits than isolated single-component interventions, possibly because of synergistic effects [85]. New technologies further broaden the therapeutic spectrum for promoting neuroplasticity, the mechanism by which the brain reorganizes after stroke to compensate for cognitive deficits (see Fig. 1 for an overview of the role of neuroplasticity in PSCI). These approaches include pharmacological strategies, noninvasive brain stimulation, cognitive training, robot-assisted therapies, and virtual reality [84,86]. A key challenge will be to tailor these interventions to individual patient characteristics in order to maximize their therapeutic potential.

### 5.3 Barriers to care and implementation challenges

Despite encouraging advances, several barriers continue to limit progress in PSCI management. These include limited resources, a shortage of trained professionals, the absence of structured care pathways, and persistent inequities in patient access to diagnosis, treatment, and follow-up care. Cognitive screening remains inconsistently embedded in post-stroke workflows, and although recent scientific statements and guidelines provide a useful framework for PSCI care, many recommendations remain constrained by limited and low-certainty evidence [6,52]. Stronger implementation strategies are needed to translate current evidence into practice and to promote more equitable access to care.

Particular attention must also be given to the urban-rural divide, which is often more pronounced in low- and middle-income countries: patients in rural areas face delayed access to emergency stroke services, diagnostic imaging, and specialized neurological or cognitive care because of geographic, infrastructural, and resource constraints [87,88].

At present, non-pharmacological approaches remain the most practical and widely applicable options for PSCI management. Their effective delivery, however, will depend on better integration of cognitive care into routine stroke pathways and on health-system strategies that can support implementation beyond specialized centers [77].

## 6 Prevention and risk management in PSCI

### 6.1 A brain health framework for prevention

While advances in diagnostic technology enable earlier and more precise detection of PSCI, emerging evidence underscores that the most effective strategy remains prevention. Beyond disease-specific diagnosis and treatment, a broader framework is needed—one that addresses modifiable risk factors across the lifespan and supports optimal brain functioning before, during, and after stroke.

The concept of brain health provides such a framework. According to the World Health Organization, brain health is not merely the absence of neurological disorders but a continuous, life-course process shaped by medical, lifestyle, and psychosocial influences [89]. It represents a holistic paradigm for cognitive protection, extending beyond disease-specific treatment to encompass a dynamic state of optimal brain functioning across cognitive, sensory, social-emotional, behavioral, and motor domains.

In the context of stroke and PSCI, this framework emphasizes cerebrovascular prevention alongside strategies that strengthen resilience and adaptive brain functioning. In population-based studies, healthier lifestyle patterns are associated with reduced dementia risk and more favorable cognitive trajectories [90,91], and evidence in stroke survivors suggests that such patterns may be linked to a slower rate of post-stroke cognitive decline [92]. Likewise, psychosocial engagement and cognitively stimulating activities—key contributors to cognitive reserve—support brain health across the lifespan [93] and may help protect against cognitive decline after stroke [94,95]. This perspective also highlights midlife as a critical window for prevention, while recognizing that later-life interventions may still modify trajectories of decline [96]. By embedding these preventive and brain health-promoting principles into stroke care and PSCI management, brain health provides a unifying framework that complements disease-focused approaches and supports long-term quality of life [97,98].

### 6.2 Prevention across the stroke continuum

The prevention of PSCI requires a multifaceted approach that combines vascular risk-factor control, reinforcement of brain resilience, and the promotion of healthy behaviors [1,64]. Ideally, preventive strategies should begin in the acute phase of stroke, when early cognitive impairment may already signal a less favorable long-term trajectory and when downstream secondary injury and pathobiological processes are still most amenable to modification [99]. As an emerging therapeutic concept, adjunctive agents administered alongside or immediately after reperfusion therapy may help limit secondary injury cascades [[100], [101], [102]]. Because stroke and cognitive impairment share overlapping pathophysiological mechanisms, strategies aimed at reducing recurrent stroke risk are also likely to mitigate the risk of cognitive decline, although the magnitude of the direct cognitive benefit remains uncertain [1,52,99].

### 6.3 Vascular risk-factor management

In discussing vascular risk factors, it is important to distinguish between prevention of stroke itself and prevention of PSCI after stroke. In stroke survivors, short- and medium-term cognitive decline does not appear to relate equally to all vascular risk factors. In particular, diabetes and post-stroke hyperglycemia warrant special attention, as higher post-stroke glucose levels have been associated with faster decline in global cognition, whereas post-stroke systolic blood pressure and LDL cholesterol have not shown the same relationship [103]. Given the rising global prevalence of diabetes, this distinction is especially relevant to PSCI prevention and risk stratification.

Hypertension nevertheless remains a central modifiable determinant of recurrent stroke and broader cerebrovascular risk. Accordingly, blood pressure control remains a core component of secondary vascular prevention after stroke, even though its specific cognitive benefits appear less consistent than those associated with post-stroke hyperglycemia [6,52]. Dietary factors such as excessive salt intake may further contribute to this risk through their effects on blood pressure [104].

More broadly, treatable vascular exposures such as diabetes and markers of cerebral small vessel disease, particularly white matter hyperintensities, are increasingly recognized as relevant targets in the secondary prevention of adverse cognitive outcomes after stroke [99]. Comprehensive vascular risk-factor management should therefore be regarded as an integral component of strategies to prevent or delay PSCI [6,52,99].

### 6.4 Lifestyle modification

Key interventions include smoking cessation, limiting alcohol intake, and maintaining a healthy weight. Smoking has well-known effects on the cardiovascular system and neurons, mediated by oxidative stress and inflammation, while heavy alcohol use is associated with increased risk of cognitive impairment [64].

Regular physical activity, encompassing both aerobic and resistance training, is an important component of secondary prevention after stroke and may also improve executive function, processing speed, and memory in stroke survivors [76,105,106], likely through enhanced cerebral blood flow, neuroplasticity, and reduced inflammation [105,107].

Similarly, adherence to the Mediterranean diet—rich in fruits, vegetables, whole grains, fish, and unsaturated fats—confers anti-inflammatory and vasoprotective effects [108,109], thereby lowering the risk of stroke [110], Alzheimer’s disease, and cognitive decline [111].

Recent evidence also underscores the importance of sleep quality as a modifiable factor. Healthy sleep patterns are associated with a reduced risk of cognitive deterioration in patients with cerebrovascular disease, highlighting sleep optimization as a promising but still underexplored target for PSCI prevention [112,113].

### 6.5 Psychosocial factors and caregiver involvement

Psychosocial factors deserve consideration in PSCI because they may influence both cognitive trajectories and the wider impact of stroke-related cognitive deficits. In a prospective study, early depressive symptoms and higher self-perceived burden were independently associated with cognitive impairment at three months after acute ischemic stroke, whereas the effect of social support appeared less consistent after multivariable adjustment [114]. Poorer post-stroke cognition has also been associated with worse stroke survivor quality of life, lower caregiver quality of life, and greater caregiver burden, underscoring the broader clinical relevance of cognitive sequelae beyond the patient alone [115]. Family- or dyad-focused psychoeducational interventions may help reduce caregiver burden and improve selected survivor outcomes, although the evidence remains heterogeneous and not all psychosocial endpoints improve consistently [116]. Accordingly, psychosocial support and active caregiver involvement should be considered complementary components of comprehensive PSCI care rather than stand-alone prevention strategies.

## 7 Future directions and unmet needs

Despite growing recognition of PSCI as a major consequence of cerebrovascular disease, substantial challenges remain in its diagnosis, management, and research. These challenges reflect the complexity and heterogeneity of PSCI and underscore the need for more precise, individualized, and better integrated approaches to care.

### 7.1 Core challenges in PSCI research and clinical practice

Three central and interconnected challenges continue to impede progress in PSCI research and clinical practice.

1. *Clinical and pathological heterogeneity*
  Patients with PSCI present with diverse cognitive profiles, stroke etiologies, and comorbidities. Differences in lesion location, age, and cognitive reserve [2,117,118] complicate outcome prediction and trial interpretation, as interventions may have variable effects across subgroups [2,117,119].
2. *Overlap with covert vascular and neurodegenerative pathologies*
  Many stroke survivors—particularly older adults—harbor covert vascular and neurodegenerative pathologies, including small vessel disease, white matter hyperintensities, and early Alzheimer-type changes. These processes can influence cognitive trajectories and complicate differential diagnosis [47,64].
3. *Methodological limitations*
  Clinical trials are often limited by the absence of standardized cognitive endpoints, inadequate stratification, and insufficient long-term follow-up [120]. Furthermore, global screening tools may lack sensitivity to the characteristic cognitive profile of vascular cognitive impairment, which is frequently marked by disproportionately greater executive dysfunction than memory impairment [42].

### 7.2 The path forward: Precision medicine and patient-centered research

Addressing these challenges will require more personalized and risk-stratified approaches to PSCI. Advances in neuroimaging, fluid biomarkers, and digital tools may improve prediction models, refine phenotyping, and support more individualized care. A precision medicine framework—stratifying patients according to imaging-defined subtypes, cognitive profiles, and underlying pathology—is likely to improve treatment selection and increase the likelihood of detecting meaningful effects in future interventional studies [1,121].

At the same time, future research should move beyond cognitive test scores alone and place greater emphasis on patient-centered outcomes. Measures such as functional independence, quality of life, return to work, and caregiver burden provide a more meaningful reflection of the real-world impact of PSCI and should play a more prominent role in defining treatment goals and evaluating intervention success [115,122].

### 7.3 Integration into clinical care

A major unmet need in PSCI care is not only the development of better interventions, but their consistent integration into routine stroke services. Despite increasing recognition of PSCI, substantial variation in diagnostic practice, follow-up, and access to cognitive care remains evident across healthcare settings. Limited specialist variability, fragmented care pathways, and the continued scarcity of high-certainty guidance all contribute to underdiagnosis, uncertainty in management, and uneven implementation of available strategies. Addressing these gaps will require more systematic embedding of cognitive assessment, longitudinal follow-up, and multidisciplinary support into stroke pathways, alongside implementation frameworks that can translate emerging evidence into routine care.

Many of the priorities identified more than a decade ago—including harmonization of diagnostic criteria, integration of prevention into routine care, and development of targeted interventional trials—therefore remain unresolved and highly relevant today [13].

### 7.4 Technology and innovation

Emerging technologies offer important opportunities to improve PSCI detection, monitoring, and management. Artificial intelligence and machine-learning approaches may enhance prediction models by integrating multimodal data from imaging, biomarkers, and clinical assessments. Digital health platforms and telemedicine may also expand access to cognitive screening and rehabilitation, particularly in underserved regions.

Wearable devices and smartphone-based tools could further support continuous monitoring of cognitive and functional change, enabling earlier recognition of decline and more personalized follow-up. Although these approaches require rigorous validation, they hold considerable promise for making PSCI care more scalable, responsive, and individualized.

### 7.5 Global perspectives and health equity

Addressing global disparities in PSCI management remains a critical priority. Low- and middle-income countries face unique challenges, including limited access to neuroimaging, shortages of trained personnel, and a lack of culturally adapted diagnostic tools. Future efforts should focus on developing scalable interventions tailored to local needs and capacity, while also strengthening health-system infrastructure.

Community-based programs such as the Austrian Vietnamese Advancement Neurorehabilitation Treatment (AVANT) program in Vietnam illustrate the potential of caregiver education and structured rehabilitation to improve outcomes even in resource-limited settings. International collaboration, context-sensitive implementation strategies, and knowledge sharing will be essential to reducing the global burden of PSCI and improving equitable access to post-stroke cognitive care.

## 8 Conclusion

Post-stroke cognitive impairment is a common and disabling consequence of cerebrovascular disease, affecting a substantial proportion of stroke survivors and profoundly impacting independence and quality of life [6]. It arises from the interaction of pre-existing vulnerability, acute lesion-related injury, and post-stroke processes—including vascular pathology, inflammation, blood-brain barrier dysfunction, and related secondary injury mechanisms—which together result in a heterogeneous clinical presentation that poses major challenges for clinicians and researchers.

Despite its high prevalence, a substantial gap remains between recognition of PSCI and its management in routine clinical care. Pharmacological therapies developed for Alzheimer's disease, such as cholinesterase inhibitors, memantine, and other neuroprotective agents, have shown limited and inconsistent benefits in stroke populations, and current evidence remains insufficient to support their routine use [52]. Cerebrolysin has emerged as a promising candidate, supported by comparative effectiveness data and ongoing randomized controlled trials such as CODEC, which may help clarify its role in post-stroke cognitive decline [74,75].

Non-pharmacological interventions—particularly cognitive rehabilitation and physical activity—show promise but remain underutilized because of systemic barriers, including limited service availability, insufficiently trained personnel, and inadequate integration of cognitive care into established stroke pathways that frequently focus on the acute disease stage only [6,52].

A major complicating factor is the frequent presence of covert vascular and neurodegenerative pathologies, including small vessel disease, silent infarcts, and preclinical Alzheimer-type changes, which may remain undetected yet strongly influence prognosis [47,64]. Failure to account for this heterogeneity in clinical trials has likely contributed to diluted treatment effects and slower progress in the field [121,123].

Moving forward, a paradigm shift toward personalized, risk-stratified management is essential. Advances in neuroimaging, fluid biomarkers, and comprehensive neuropsychological profiling provide powerful tools to identify high-risk individuals and tailor interventions to their specific pathological and cognitive profiles [1,121]. Future clinical trials must adopt such stratification methods to enhance therapeutic precision.

Outcome measures should also expand beyond cognitive test scores to include patient-centered domains such as functional independence, quality of life, and caregiver burden, as these better capture the real-world impact of PSCI [115,122]. Ultimately, the greatest opportunity to reduce the long-term burden of PSCI lies in prevention, particularly through vascular risk reduction and the promotion of physical, cognitive, and social engagement [120,124]. At the same time, integrating cognitive health into comprehensive stroke care—through targeted assessment, multidisciplinary management, and patient-centered planning—should be regarded as a key priority for routine stroke services [6,52].

## Declaration of generative AI and AI-assisted technologies in the manuscript preparation process

During the preparation of this work, the authors used ChatGPT 4o, 5, 5.1, 5.2, and 5.4 Thinking along with Manus (powered by Claude 3.7), Manus 1.5 and 1.5 Lite to generate a preliminary outline for the literature review, conduct the literature search, summarize articles, and enhance the language and readability of the manuscript. After using this service, the authors reviewed and edited the content as needed and take full responsibility for the content of the published article.

## CRediT authorship contribution statement

**Leonardo Pantoni:** Writing – review & editing, Conceptualization. **Slaven Pikija:** Writing – original draft, Project administration, Conceptualization. **Tuyen Van Nguyen:** Writing – review & editing. **Michael Brainin:** Writing – review & editing, Conceptualization.

## Declaration of competing interest

The authors declare the following financial interests/personal relationships which may be considered as potential competing interests: Leonardo Pantoni reports a relationship with Medtronic, Piam Pharmaceuticals, Amicus, Boston Scientific Corporation that includes: consulting or advisory. Slaven Pikija reports a relationship with EVER Pharma, Medtronic, Lilly that includes: speaking and lecture fees. Michael Brainin reports a relationship with EVER Pharma that includes: consulting or advisory and speaking and lecture fees. Given his role as Editorial Board Member of CCCB, Leonardo Pantoni had no involvement in the peer review of this article and had no access to information regarding its peer review. Full responsibility for the editorial process for this article was delegated to another journal editor. If there are other authors, they declare that they have no known competing financial interests or personal relationships that could have appeared to influence the work reported in this paper.
